# Modulation of Bi*_x_*Sb_2−*x*_Te_3_ Alloy Application Temperature via Optimizing Material Composition

**DOI:** 10.3390/ma17235751

**Published:** 2024-11-24

**Authors:** Shifang Ma, Jianan Li, Daming Du, Xuefeng Ruan, Ming Cao, Ming Lin, Qiongxin Hua, Qi Luo, Ping Tang, Jinzhao Guan, Jian Yu

**Affiliations:** 1 School of Materials Science and Engineering, Jiujiang University, Jiujiang 332005, China; jiananli0303@163.com (J.L.); 6100072@jju.edu.cn (D.D.); cm0405@126.com (M.C.); linmingzny@163.com (M.L.); huaqx_39@126.com (Q.H.); 6100092@jju.edu.cn (Q.L.); tangping_101@163.com (P.T.); guanjinzhao@163.com (J.G.); 2 Jiangxi Key Laboratory of Material Surface Engineering, Jiujiang University, Jiujiang 332005, China; 3 School of Power and Mechanical Engineering, Wuhan University, Wuhan 430072, China; xf-ruan@whu.edu.cn; 4 Nanjing Institute of Geography and Limnology, Chinese Academy of Sciences, Nanjing 210008, China

**Keywords:** Bi*_x_*Sb_2−*x*_Te_3_, application temperature, band gap, thermoelectric performance

## Abstract

Bi_2_Te_3_-based alloys are representatively commercialized thermoelectric materials for refrigeration and power generation. Refrigeration mainly utilizes thermoelectric properties near room temperature, while the power generation temperature is relatively high. However, it is difficult for bismuth telluride to maintain good thermoelectric properties throughout the entire temperature range of 300–500 K. Herein, a series of Bi*_x_*Sb_2−*x*_Te_3_ alloys with different Bi contents were prepared by a simple preparation method and systematically investigated, and their best application temperature range was found. The Bi content can modulate carrier concentration and band gap, and the maximum dimensionless figure of merit (*ZT*) value of Bi*_x_*Sb_2−*x*_Te_3_ can be achieved in the corresponding application temperature range. The maximum *ZT* of Bi_0.3_Sb_1.7_Te_3_ with a Bi content equal to 0.3 reaches 1.14 at 400 K, and the average *ZT* is 1.06 in the range of 300–500 K, which is suitable for both power generation and refrigeration. Therefore, power generation technologies with higher application temperatures should be selected from Bi*_x_*Sb_2−*x*_Te_3_ materials with Bi content less than 0.3, and refrigeration technologies with lower application temperatures should be selected with Bi content greater than 0.3. This work provides experimental guidance for finding the composition of Bi_2_Te_3_-based alloys in scientific research and practical applications.

## 1. Introduction

Thermoelectric (TE) materials can directly mutually convert heat–electricity energy without any harmful substance emissions [1,2], which provides an eco-friendly pathway and promising applications for all solid-state refrigeration and power generation techniques based on the Peltier effect and Seebeck effect. The TE device conversion efficiency is closely related to the dimensionless figure of merit, *ZT* = *α*^2^*σT*/*κ*, where *α*^2^*σ*, *T*, and *κ* are the power factor (including Seebeck coefficient *α* and electrical conductivity *σ*), absolute temperature, and thermal conductivity, respectively [3,4,5]. According to the above formula, the performance of materials is proportional to the power factor and inversely proportional to the thermal conductivity. Therefore, the ideal TE materials require a relatively larger *α*^2^*σ* and lower *κ* [6,7]. Due to the intrinsically intricate coupling connection between the aforementioned parameters, the improvement of TE performance is a critical challenge [8,9]. In addition, a simple and high-efficiency synthesis in terms of economy is also crucial for practical commercial applications.

Bi_2_Te_3_ and related alloys are the most extensively studied TE materials and the only ones with commercial applications below 500 K. Among the numerous Bi_2_Te_3_ alloys, the zone-melting Bi_2_Te_3_ with a maximum *ZT* value of around 1.0 in approximately room temperature conditions is widely applied in TE refrigeration and waste heat recovery [10,11,12]. However, zone-melting Bi_2_Te_3_ has two disadvantages. For one thing, the weak van der Waals bonding between adjacent atom layers leads to inferior mechanical performance, resulting in material splitting during the cutting process and material waste. For another, the zone-melted Bi_2_Te_3_ maximum *ZT* is about 1.0 and occurs near room temperature, which is relatively low and limits their further commercial applications. Therefore, extensive endeavors have been devoted to heightening the TE properties and broadening the application temperature range of polycrystalline Bi_2_Te_3_. In the past several decades, carrier engineering [13,14,15] and nanostructure engineering [16,17,18] have been applied to control carrier and phonon transport properties, thus improving the TE performance of polycrystalline Bi_2_Te_3_. For p-type Bi_2_Te_3_ alloys, in particular, dense bulks with a maximum *ZT* value above 1.2 have been prepared by hot deformation [19,20,21], three-dimensional printing [22,23], melt spinning [24,25], cyclic SPS [26], liquid-phase sintering [27,28,29], or solvothermal synthesis [30,31]. Although the TE performance of polycrystalline p-type Bi_2_Te_3_ obtained by the above methods is better than that of zone-melted materials, it requires a long preparation time, complex operation, complex preparation equipment, or difficult synthesis control, which limits its commercial application.

Ball milling is a simple, well-known, and established preparation technique for obtaining Bi_2_Te_3_ powders and is recognized as an indispensable technology for synthesizing TE materials. The obtained p-type polycrystalline Bi_2_Te_3_ bulk performance is better than the TE performance achieved using the zone-melted method; the maximal *ZT* value reaches 1.4 at 373 K due to the enhanced phonon scattering caused by nanocrystallinity and a large reduction in the lattice thermal conductivity [32]. Subsequently, many studies have focused on ball milling to improve the TE properties of p-type Bi_2_Te_3_ alloys [33,34,35]. Although those materials possess high TE performance, there is still a key issue that needs further exploration: the bulk materials’ optimal composition for different application temperatures in the range of 300–500 K. The materials’ composition affects the formation of the antisite defect, which is closely related to the electrical transport properties of the material. Optimizing electrical transport properties is an effective measure to modulate the application temperature range of materials. This investigation focuses on p-type Bi_2_Te_3_-based alloys, so Bi, Sb, and Te were selected for the composition.

In this work, a series of p-type Bi*_x_*Sb_2−*x*_Te_3_ bulk materials were prepared by a simple preparation method (ball milling and spark plasma sintering). The phase composition and TE properties of these bulk materials have been systematically investigated. The Bi content has a great influence on the electrical and thermal transport properties. As a result, the modulation of the Bi content can effectively adjust the optimal application temperature range of p-type Bi*_x_*Sb_2−*x*_Te_3_. Although TE materials are slightly lower than the above methods, the p-type Bi*_x_*Sb_2−*x*_Te_3_ with an adjustable application temperature range is more beneficial to promoting large-scale commercial applications.

## 2. Experimental Section

High-purity Bi (99.99%), Sb (99.99%), and Te (99.99%) powders were mixed according to the stoichiometric composition of *p*-type Bi*_x_*Sb_2−*x*_Te_3_ (*x* = 0.2, 0.3, 0.4, 0.5, 0.6). The mixed powders were loaded into stainless-steel jars with stainless-steel balls. The jars were vacuum-sealed and filled with an argon gas mixture three times, and then set on a planetary ball-milling machine for 360 min at 450 rpm in an argon-protective atmosphere. The synthesized powders were loaded into a graphite die for spark plasma sintering (SPS) at 673 K with an axial pressure of 50 MPa for 5 min. 

The phase purity and composition of synthesized *p*-type Bi*_x_*Sb_2−*x*_Te_3_ materials were analyzed by X-ray diffraction (XRD, Cu *Kα*, Bruker D8, Selb, Germany). The surface morphology and elemental distribution of the *p*-type Bi*_x_*Sb_2−*x*_Te_3_ bulk were investigated by an electron probe micro analyzer (EPMA) (JEOL, JXA-8230, Kyoto, Japan). The rectangular specimens (about 2.5 × 3.5 × 11.5 mm) were cut from the sintered bulk materials and were used to measure electrical conductivity *σ* and Seebeck coefficient *α* by the four-probe method (Cryoall CTA-3/500). The thermal conductivity *κ* was calculated using the formula *κ* = *DρC*_p_. The disk specimens (about *ϕ* 6 × 1.5 mm) were cut for thermal diffusivity *D* measurement, which were measured by a Netzsch laserflash instrument (LFA-467, Selb, Germany). The sample density *ρ* was obtained by the Archimedes method. The specific heat capacity *C*_p_ is estimated by Dulong–Petit law. The carrier thermal conductivity was estimated by the Wiedemann–Franz law of *κ_E_* = *σLT*, where *L* is the Lorentz number. The lattice thermal conductivity *κ_L_* was calculated according to the equation *κ_L_* = *κ* − *κ_E_*. The Hall coefficient *R_H_* from 300 K to 500 K was collected on a self-made test system with the magnetic field varied in the range of ±2.0 T. The carrier concentration *n_H_* and Hall mobility *μ_H_* were calculated according to the equations *n_H_* = 1/(*eR_H_*) and *μ_H_* = *σ*/(*en_H_*), respectively. 

## 3. Results and Discussion

### 3.1. Composition and Microstructures Analysis

**Figure 1:**
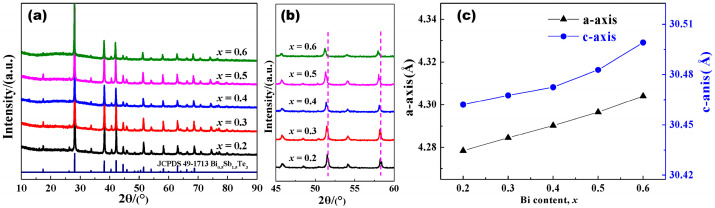
(**a**) XRD patterns of sintered Bi*_x_*Sb_2−*x*_Te_3_ materials. (**b**) The expanded view of XRD in the range of 45–60°. (**c**) Lattice parameters of sintered Bi*_x_*Sb_2−*x*_Te_3_ materials.

The purity and lattice parameters of the sintered Bi*_x_*Sb_2−*x*_Te_3_ (*x* = 0.2, 0.3, 0.4, 0.5, 0.6) polycrystalline materials were analyzed by XRD. As shown in Figure 1a, the major diffraction peaks of Bi*_x_*Sb_2−*x*_Te_3_ materials can be well indexed to the standard pattern of JCPDS 49–1713, indicating that the main phase of the sintered materials is the Bi_0.5_Sb_1.5_Te_3_ phase with the rhombohedral structure. In order to analyze the influence of Bi content on phase structure, the XRD patterns of the sintered Bi*_x_*Sb_2−*x*_Te_3_ materials were expanded in the range of 45–60° (Figure 1b). Obviously, the diffraction peaks near 51° and 58° move to a lower angle with an increase in Bi content. According to the Bragg equation 2*dsinθ* =*λ* (where *d*, *θ*, and *λ* are interplanar distance, Bragg angle, and XRD wavelength, respectively), when the *λ* remains unchanged, *θ* decreases and *d* increases, indicating that the lattice expands with increasing Bi contents. The lattice parameters of Bi*_x_*Sb_2−*x*_Te_3_ were calculated according to XRD results (Figure 1c). With an increase in Bi replacing Sb content, the size along the a-axis and c-axis of the Bi*_x_*Sb_2−*x*_Te_3_ unit cell increases gradually, resulting in an expansion of the lattice parameters along both directions. The lattice expansion is attributed to the larger atomic radius of Bi (R_Bi_ = 1.55 Å) in comparison with that of Sb (R_Bi_ = 1.45 Å). The above results indicate that all Bi*_x_*Sb_2−*x*_Te_3_ alloys with different Bi contents are single-phase materials, and the Bi element successfully occupies the Sb site in the crystal structure, which is beneficial to explain the influence of Bi content on electrical and thermal transport properties of Bi*_x_*Sb_2−*x*_Te_3_.

**Figure 2:**
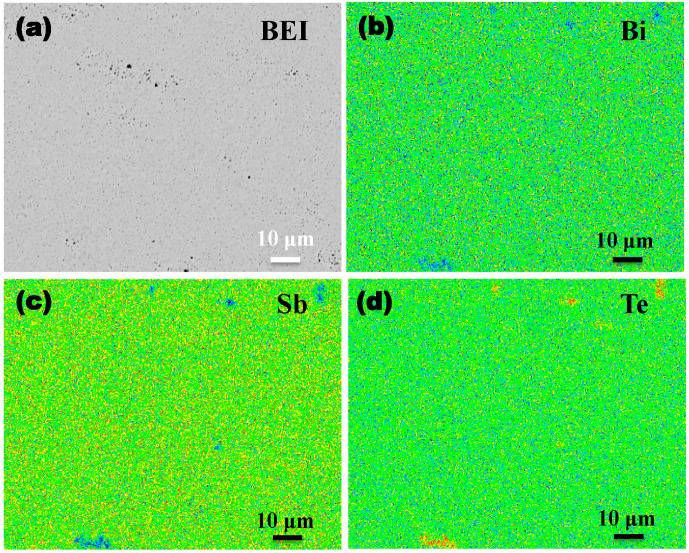
(**a**) Backscattered electron image of Bi_0.4_Sb_1.6_Te_3_. (**b**–**d**) WDS elemental mapping of Bi, Sb, and Te elements, respectively.

As shown in Figure 2a, the backscattered electron image of the Bi_0.4_Sb_1.6_Te_3_ material contains many micropores (black contrast area), which will be further analyzed in the following macrostructure analysis. Immediately afterwards, an electron probe micro analyzer (EPMA) equipped with wave-dispersive spectrometry (WDS) was utilized to investigate the elements’ distributions of Bi_0.4_Sb_1.6_Te_3_ materials. Except for some distinct shape and size areas, the Bi, Sb, and Te elements’ distributions are uniformly distributed in Bi_0.4_Sb_1.6_Te_3_ materials (Figure 2b–d). By comparing the distribution of the three elements, it was found that Bi and Sb are absent in these positions, while Te is enriched in the same position, revealing that the distinct shape and size areas are a Te impurity phase. However, no characteristic diffraction peaks of Te were detected in XRD results due to the low content, which was far less than the detection limit of XRD technology (about 1%). The analysis indicates that sintered materials are composed of the Bi*_x_*Sb_2−*x*_Te_3_ main phase and the Te impurity phase.

**Figure 3:**
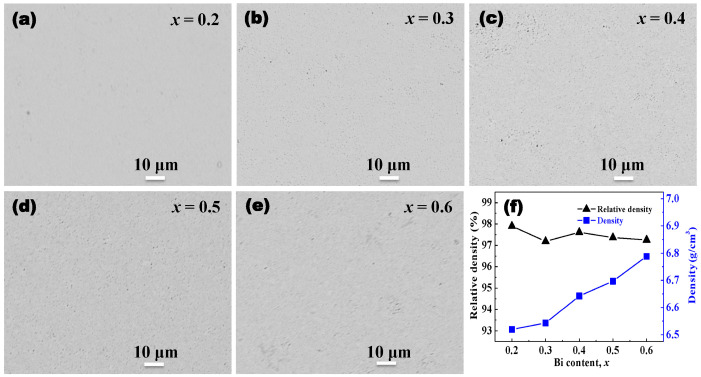
(**a**–**e**) Backscattered electron images of Bi*_x_*Sb_2−*x*_Te_3_ materials. (**f**) Density of Bi*_x_*Sb_2−*x*_Te_3_ materials.

The backscattered electron images of all the sintered Bi*_x_*Sb_2−*x*_Te_3_ bulk polished surfaces were investigated by EPMA. It can be seen that a lot of black contrast is distributed over the gray contrast, where the black contrast and the gray contrast areas are micropores and Bi*_x_*Sb_2−*x*_Te_3_ (Figure 3a–e), respectively. There are two main causes for forming micropores in bulk materials. One is that the adsorbed oxygen was not completely discharged during the sintering process, leading to the formation of micropores inside the bulk materials. Another is that liquefied Te was partially extruded, and then left many internal voids. A similar phenomenon was also reported in some studies in the literature [33,34,36]. Figure 3f shows the density and relative density of all the sintered Bi*_x_*Sb_2−*x*_Te_3_. The density gradually increases with an increase in the Bi/Sb ratio due to the larger relative atomic mass of Bi than Sb. However, the relative density fluctuates between 97.2% and 97.9%, maintaining a small range of change, indicating that the formation of micropores during the sintering process is not significantly affected by the Bi/Sb ratio. All the Bi*_x_*Sb_2−*x*_Te_3_ bulk materials contain about 2.5% micropores, which can enhance the phonon scattering and is beneficial in regulating phonon transport.

### 3.2. Thermoelectric Properties

**Figure 4:**
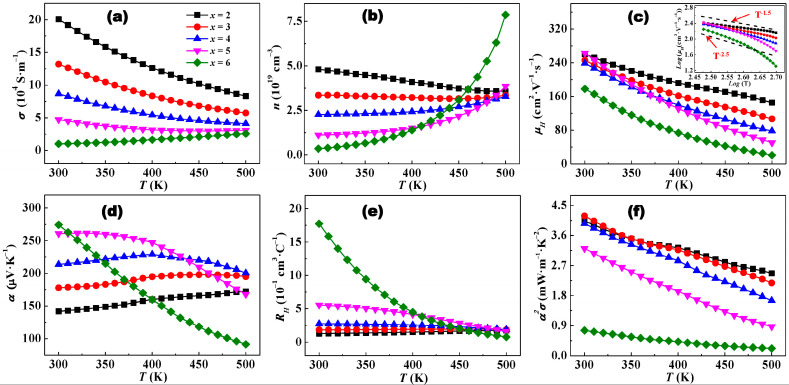
Temperature dependencies of (**a**) electrical conductivity, (**b**) carrier concentration, (**c**) Hall mobility, (**d**) Seebeck coefficient, (**e**) Hall coefficient, and (**f**) a power factor for Bi*_x_*Sb_2−*x*_Te_3_ materials. The inset in (**c**) is log(*μ*_H_) versus log(T).

The temperature-dependent electrical transport performances are presented for Bi*_x_*Sb_2−*x*_Te_3_ (*x* = 0.2, 0.3, 0.4, 0.5, 0.6) materials in Figure 4. As shown in Figure 4a, the electrical conductivity *σ* significantly decreases with increasing Bi content at room temperature and slightly reduces at 500 K. The *σ* of Bi*_x_*Sb_2−*x*_Te_3_ in the range of 0.2 ≤ *x* ≤ 0.5 monotonously decreases as the temperature increases from 300 K to 500 K, consistent with typical metallic behavior. The *σ* of Bi*_x_*Sb_2−*x*_Te_3_ with *x* = 0.6 shows an opposite change in regularity, implying a typical semiconductor behavior. Therefore, the carrier concentration *n_H_* and Hall mobility *μ_H_* were measured to uncover the opposite and inconsistent behavior. With increasing Bi content from 0.2 to 0.6, the room temperature *n_H_* decreases gradually from 4.81 × 10^19^ cm^−3^ to 0.35 × 10^19^ cm^−3^, which results from the change in defects in the Bi_2_Te_3_ alloy (Figure 4b). In fact, the antisite defects (${\mathrm{Sb}}_{\mathrm{Te}}^{\prime }$ and ${\mathrm{Bi}}_{\mathrm{Te}}^{\prime }$) and vacancy ($V_{\mathrm{Te}}^{\cdot \cdot }$) are the dominant defects in p-type polycrystalline Bi*_x_*Sb_2−*x*_Te_3_. The formation energy of antisite defect (Equation (1)) and vacancy (Equation (2)) has been reported in a previous publication [37].

(1) $$E_{\mathrm{AS}}(\mathrm{Sb}-\mathrm{Te})<E_{\mathrm{AS}}(\mathrm{Bi}-\mathrm{Te})<E_{\mathrm{AS}}(\mathrm{Sb}-\mathrm{Se})<E_{\mathrm{AS}}(\mathrm{Bi}-\mathrm{Se})$$

(2) $$E_{V}(\mathrm{Sb}-\mathrm{Te})>E_{V}(\mathrm{Bi}-\mathrm{Te})>E_{V}(\mathrm{Sb}-\mathrm{Se})>E_{V}(\mathrm{Bi}-\mathrm{Se})$$

Obviously, the formation energy of the ${\mathrm{Bi}}_{\mathrm{Te}}^{\prime }$ antisite defect is larger than that of the ${\mathrm{Sb}}_{\mathrm{Te}}^{\prime }$ antisite defect; thus, the concentration of antisite defects will decrease with increasing Bi content. The formation energy of vacancy reduces with the substitution of Sb by Bi, which boosts the vacancy formation. The antisite defect generates holes, and vacancy generates electrons. It is well known that the majority carrier of p-type Bi_2_Te_3_ alloy is holes, so *n_H_* is reduced with increasing Bi content at room temperature.

As shown in Figure 4b, the variation trend of *n_H_* over the entire temperature range is inconsistent. To uncover the variation trend, the band gaps (*E_g_*) were estimated according to Equation (3):

*E_g_* = 2*eα*_max_*T*_max_ (3)

where *α*_max_ and *T*_max_ are the maximum value of the Seebeck coefficient and the corresponding temperature, respectively. The estimated calculation results are listed in Table 1. The estimated *E_g_* values are decreased from 0.20 eV to 0.17 eV for the Bi*_x_*Sb_2−*x*_Te_3_ sample (*x =* 0.3, 0.4, and 0.5). For the Bi_0.2_Sb_1.8_Te_3_ sample, *α*_max_ is achieved at 500 K and still increases with the testing temperature (Figure 4d). For the Bi_0.6_Sb_1.4_Te_3_ sample, *α*_max_ is achieved at 300 K, and an even larger value may be obtained if the testing temperature is below 300 K. The *E_g_* values of Bi_0.2_Sb_1.8_Te_3_ and Bi_0.6_Sb_1.4_Te_3_ can be rationally deduced. The *E_g_* values of Bi_0.2_Sb_1.8_Te_3_ will be larger than 0.20 eV and the *E_g_* values of Bi_0.6_Sb_1.4_Te_3_ will be lower than 0.17 eV. *E_g_* is the minimum energy for the carrier to transition from the valence band to the conduction band. If *E_g_* is very low, carriers are more likely to be excited as the temperature increases, resulting in an intrinsic excitation. The diminished *E_g_* values explicitly give a reasonable explanation for *n_H_* increasing above room temperature. Therefore, the *n_H_* of the Bi*_x_*Sb_2−*x*_Te_3_ sample (*x =* 0.4, 0.5, and 0.6) gradually rises with testing temperature increases.

The *μ_H_* values of the Bi*_x_*Sb_2−*x*_Te_3_ samples are almost constant or slightly decreased except for the Bi_0.6_Sb_1.4_Te_3_ sample (Figure 4c). However, *μ_H_* changes significantly with improving testing temperature, indicating that the carrier scattering mechanism has changed. As shown in the inset in Figure 4c, the trend of *μ_H_* versus temperature exhibits a nearly *T*^−1.5^ exponential relationship in all testing temperatures for Bi_0.2_Sb_1.8_Te_3_, indicating that the carrier scattering mechanism is dominated by acoustic phonon scattering. The exponential relationship gradually decreases with increasing Bi content and reaches nearly *T*^−2.5^ for Bi_0.6_Sb_1.4_Te_3_, which should stem from the intrinsic excitation effect due to the decreasing *E_g_*. *n_H_* and *μ_H_* analyses can reasonably explain the *σ* change regularity.

The positive Seebeck coefficient *α* over the entire temperature range indicates that the dominant charge carriers of all samples are holes (i.e., *p*-type conduction behavior) (Figure 4d), which is consistent with the Hall coefficient (Figure 4e). At room temperature, the *α* of Bi*_x_*Sb_2−*x*_Te_3_ significantly increases with the increasing Bi content except for Bi_0.6_Sb_1.4_Te_3_. As is well known, the *α* of the degenerate semiconductor can be expressed as Equation (4) (Mott equation):

(4) $$\alpha =\frac{8\pi^{2}k_{B}^{2}Tm^{*}}{3eh^{2}}{\left(\frac{\pi }{3n}\right)}^{\frac{2}{3}}$$

where *k_B_* is the Boltzmann constant, m* is the carrier effective mass, *e* is the elementary charge, *h* is the Planck constant, and *n* is the hole concentration for the p-type Bi_2_Te_3_ alloy. The m* at room temperature was calculated using the same method in our previously reported study (Table 1) [38]. According to the Mott equation, *α* is proportional to m* and inversely proportional to *n_H_*. The decreased *n_H_* is beneficial to *α* and the decreased m* is disadvantageous to *α*. Therefore, compared with Bi*_x_*Sb_2−*x*_Te_3_ (*x* = 0.2, 0.3, 0.4, and 0.5), the less increase in *α* of Bi_0.6_Sb_1.4_Te_3_ stems from a significant decrease in m*, which may be related to the change in band structure caused by Bi content.

The *α* of Bi*_x_*Sb_2−*x*_Te_3_ (*x =* 0.2, 0.3, 0.4, 0.5, and 0.6) samples exhibit the dissimilarity trend with increasing temperature. The *α* of Bi_0.2_Sb_1.8_Te_3_ gradually rises with the increase in temperature and Bi_0.6_Sb_1.4_Te_3_ shows an inverse trend. For Bi*_x_*Sb_2−*x*_Te_3_ (*x* = 0.3, 0.4, and 0.5), *α* initially increases, reaching its maximum value, then decreases in the entire temperature range. Due to the gradual decreases in *E_g_* and *n_H_*, the temperature of maximum *α* shifts gradually to a lower temperature, mainly originating from the activation of the effect of the minority carrier. With increasing Bi content, the power factor *α*^2^*σ* firstly rises and then falls due to the combined effect of *α* and *σ* (Figure 4f). For the Bi_0.3_Sb_1.7_Te_3_ sample, the highest *α*^2^*σ* value reaches 4.2 mW·m^−1^·K^−2^ at 300 K and gradually decreases with increasing temperature.

**Figure 5:**
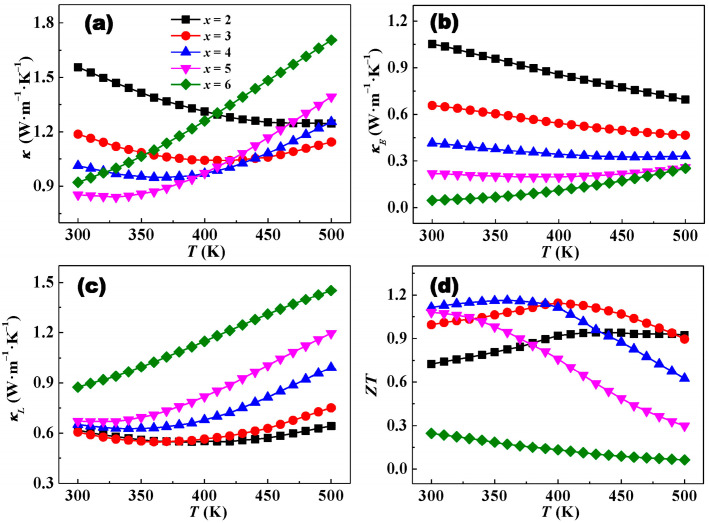
Temperature dependence of (**a**) thermal conductivity, (**b**) carrier thermal conductivity, (**c**) lattice thermal conductivity, and (**d**) *ZT* for Bi*_x_*Sb_2−*x*_Te_3_ materials.

The thermal transport performance and dimensionless figure of merit (*ZT*) values of Bi*_x_*Sb_2−*x*_Te_3_ (*x* = 0.2, 0.3, 0.4, 0.5, and 0.6) specimens are depicted in Figure 5. The thermal conductivity *κ* firstly drops and then rises with increasing Bi content at room temperature (Figure 5a). The Bi_0.5_Sb_1.5_Te_3_ sample has the lowest *κ* and is about 0.85 W·m^−1^·K^−1^ at 300 K, a 46% reduction as compared with that of Bi_0.2_Sb_1.8_Te_3_. The *κ* change trend of Bi*_x_*Sb_2−*x*_Te_3_ with the increasing temperature is related to the evolutions of *E_g_* and *n_H_*, which will be discussed later. The above results indicate that the Bi content can effectively impact the thermal transport properties. The Lorenz number was calculated using the same method in our pioneer work (Table 1) [39], and the obtained *κ_E_* is shown in Figure 5b. It can be seen that *κ_E_* gradually decreases with increasing test temperature, and the change trend is consistent with the change in *σ*. The lattice thermal conductivity *κ_L_* was calculated by subtracting *κ_E_* from *κ*. *κ_L_* initially decreases and then increases with increasing Bi content at room temperature (Figure 5c). There are three aspects affecting the room temperature *κ_L_*. Firstly, the enhancing cation site disorder degree with increasing Bi content results in a stronger mass and strain field fluctuation, and a decrease in *κ_L_*. Secondly, the increasing formation energy antisite defect leads to a decrease in the concentration of antisite defect with increasing Bi content, which would weaken the defect phonon scattering, and *κ_L_* will rise. Thirdly, a reduction in *n_H_* with an increase in Bi content would weaken the carrier–phonon scattering, giving rise to an increase in *κ_L_*. The competition among the three mechanisms leads to the change trend of *κ_L_* with increasing Bi content at room temperature. As the temperature increases, the *κ_L_* of Bi*_x_*Sb_2−*x*_Te_3_ (*x* = 0.2, 0.3, 0.4, and 0.5) initially decreases due to the enhanced Umklapp phonon scattering and then increases owing to the intensified intrinsic excitation. Noteworthily, the temperature of intrinsic excitation gradually shifts to a lower temperature with increasing content of Bi, which is derived from the decreases in *E_g_* and *n_H_*. The *κ_L_* of Bi_0.6_Sb_1.4_Te_3_ gradually increases with an increase in temperature, indicating that the intrinsic excitation temperature is lower than the room temperature.

The temperature-dependent *ZT* values of Bi*_x_*Sb_2−*x*_Te_3_ (*x* = 0.2, 0.3, 0.4, 0.5, and 0.6) specimens are shown in Figure 5d. The *ZT* value initially increases and then decreases with increasing Bi content at room temperature, which displays an opposite variation trend of *κ*. As is known, TE materials can be used for refrigeration and power generation, and the corresponding utilization temperatures are not similar. Refrigeration requires a large *ZT* value near room temperature and power generation needs a large *ZT* value at higher temperatures because the high device conversion efficiency requires a large ZT value. The maximum *ZT* value corresponds to the pivotal temperature shift to lower temperatures with increasing Bi content. The corresponding maximum *ZT* values for Bi*_x_*Sb_2−*x*_Te_3_ (*x* = 0.2, 0.3, 0.4, 0.5, and 0.6) specimens are 0.94 at 430 K, 1.14 at 400 K, 1.16 at 360 K, 1.08 at 300 K, and 0.24 at 300 K, respectively. Therefore, the power generation materials should be sought from Bi content near 0.2 (Bi_0.2_Sb_1.8_Te_3_) and 0.3 (Bi_0.3_Sb_1.7_Te_3_). The refrigeration materials should be sought from Bi content near 0.4 (Bi_0.4_Sb_1.6_Te_3_) and 0.5 (Bi_0.5_Sb_1.5_Te_3_). The highest average *ZT* values of Bi_0.2_Sb_1.8_Te_3_ and Bi_0.3_Sb_1.7_Te_3_ for power generation reached 0.93 and 1.05 in the range of 400–500 K. The highest average *ZT* values of Bi_0.4_Sb_1.6_Te_3_ and Bi_0.5_Sb_1.5_Te_3_ for refrigeration reached 1.14 and 0.96 in the range of 300–400 K. The *ZT* of this work approaches or exceeds the commercial zone-melting Bi_2_Te_3_ with a *ZT* value around 1.0 in approximately room temperature conditions. In particular, the highest average *ZT* of Bi_0.3_Sb_1.7_Te_3_ reaches 1.06 in the range of 300–500 K, which can be used for both refrigeration and power generation over the entire temperature range. Consequently, researchers can select suitable Bi_2_Te_3_-based material components based on the above analysis and further optimize them to obtain high-performance TE materials based on the corresponding utilization temperature range of the materials.

## 4. Conclusions

A series of Bi*_x_*Sb_2−*x*_Te_3_ (*x* = 0.2, 0.3, 0.4, 0.5, and 0.6) materials were prepared via ball milling and spark plasma sintering. The influence of Bi content on the phase composition and TE performance was systematically investigated between 300 and 500 K. The cell parameters of Bi*_x_*Sb_2−*x*_Te_3_ increase gradually with an increase in Bi replacing Sb content. The changing Bi content can optimize *n_H_*, increase *α*, greatly reduce *κ*, and adjust *E_g_*. By modulating *n_H_* and *E_g_*, the maximum *ZT* value of Bi*_x_*Sb_2−*x*_Te_3_ can be achieved in the corresponding application temperature range. Power generation technologies with higher application temperatures should be selected from Bi*_x_*Sb_2−*x*_Te_3_ materials with a Bi content of less than 0.3. Refrigeration technologies with lower application temperatures should be selected with Bi contents greater than 0.3. In particular, the maximum *ZT* of Bi_0.3_Sb_1.7_Te_3_ with a Bi content equal to 0.3 reaches 1.14 at 400 K, and the average *ZT* is 1.06 in the range of 300–500 K, which is suitable for both power generation and refrigeration. Therefore, the Bi_0.3_Sb_1.7_Te_3_ material is the most effective alloy over the entire temperature range. This work provides experimental guidance for finding the composition of Bi_2_Te_3_-based alloys in scientific research and practical applications.

## Figures and Tables

**Table 1:** Hall mobility, carrier concentration, Seebeck coefficient, carrier effective mass, band gap, and Lorenz number of Bi*_x_*Sb_2−*x*_Te_3_ at room temperature.

Samples	*μ_H_* (cm^2^/V·s)	*n_H_* (10^19^ cm^−3^)	*α* (μV/K)	m*/*m*_0_	*E_g_* (eV)	*L* (10^−8^ W·Ω/K^2^)
Bi_0.2_Sb_1.8_Te_3_	260.44	4.81	141.79	1.05	-	1.75
Bi_0.3_Sb_1.7_Te_3_	245.80	3.35	178.00	1.15	0.20	1.66
Bi_0.4_Sb_1.6_Te_3_	238.62	2.27	213.64	1.21	0.18	1.60
Bi_0.5_Sb_1.5_Te_3_	262.48	1.12	260.96	1.12	0.17	1.55
Bi_0.6_Sb_1.4_Te_3_	177.97	0.35	274.38	0.58	-	1.54

## Data Availability

The original contributions presented in the study are included in the article, further inquiries can be directed to the corresponding authors.
